# Bioleaching of Chalcopyrite Waste Rock in the Presence of the Copper Solvent Extractant LIX984N

**DOI:** 10.3389/fmicb.2022.820052

**Published:** 2022-03-18

**Authors:** Xiaohui Liu, Yuhong Li, Jianfeng Cao, Zhenshun Zeng, Xiaorong Liu, Ruiyong Zhang, Qian Li, Wolfgang Sand

**Affiliations:** ^1^Key Laboratory for Water Quality and Conservation of the Pearl River Delta, Ministry of Education, School of Environmental Science and Engineering, Guangzhou University, Guangzhou, China; ^2^Biofilm Centre, Aquatische Biotechnologie, Universität Duisburg-Essen, Essen, Germany; ^3^School of Materials Science and Engineering, Shanghai Institute of Technology, Shanghai, China; ^4^Key Laboratory of Marine Environmental Corrosion and Biofouling, Institute of Oceanology, Qingdao, China; ^5^Open Studio for Marine Corrosion and Protection, Pilot National Laboratory for Marine Science and Technology, Qingdao, China; ^6^College of Environmental Science and Engineering, Donghua University, Shanghai, China; ^7^Interdisciplinary Ecological Centre, TU Bergakademie Freiberg, Freiberg, Germany

**Keywords:** bioleaching, chalcopyrite waste rock, LIX984N, *Acidithiobacillusferrooxidans*, *Leptospirillum ferrooxidans*, *Sulfobacillus thermosulfidooxidans*

## Abstract

Heap bioleaching, the solubilization of metal ions from metal sulfides by microbial oxidation, is often combined with solvent extraction (SX) and electrowinning to recover, e.g., copper from low-grade ores. After extraction, the leaching solution is recycled, but the entrained organic solvents may be toxic to the microorganisms. Here *Acidithiobacillus ferrooxidans*, *Leptospirillum ferrooxidans*, and *Sulfobacillus thermosulfidooxidans* were selected to perform bioleaching of chalcopyrite waste rock in the presence of the SX reagent (2.5% v/v LIX984N in kerosene). Possibly inhibitory effects have been evaluated by copper extraction, bacterial activity, number of actively Fe(II)-oxidizing cells, and biofilm formation. Microcalorimetry, most probable number determination, and atomic force microscopy combined with epifluorescence microscopy were applied. The results show that 100 and 300 mg/L SX reagent could hardly inhibit *At. ferrooxidans* from oxidizing Fe^2+^, but they seriously interfered with the biofilm formation and the oxidization of sulfur, thereby hindering bioleaching. *L. ferrooxidans* was sensitive to 50 mg/L SX reagent, which inhibited its bioleaching completely. *Sb. thermosulfidooxidans* showed different metabolic preferences, if the concentration of the SX reagent differed. With 10 mg/L LIX984N *Sb. thermosulfidooxidans* preferred to oxidize Fe^2+^ and extracted the same amount of copper as the assay without LIX984N. With 50 mg/L extractant the bioleaching stopped, since *Sb. thermosulfidooxidans* preferred to oxidize reduced inorganic sulfur compounds.

## Introduction

Copper is of great importance for human society, since it has been used in virtually all branches of mature industrial or more newly industrializing economies, such as construction, transport, telecom, and all kinds of electrical and electronic appliances (Radetzki, 2009). There is no doubt that the demand for copper will keep growing. However, high-grade copper ores are almost depleted. Thus, mining industries have to recover copper from low-grade ores that used to be considered as waste. By considering high costs of traditional processes and growing concern for the environment, mining industries finally increasingly use bioleaching combined with solvent extraction (SX) and electrowinning (BL-SX-EW) to recover and produce copper from low-grade ores (Songrong et al., 2002; Panda et al., 2015; Gentina and Acevedo, 2016).

Bioleaching, the microbial dissolution of metal sulfides, is caused by Fe^3+^ ions as the crucial oxidizing reagent. Generally, after attack by Fe^3+^ ions, metal cations and reduced inorganic sulfur compounds (RISCs) are released from the minerals causing Fe^3+^ to be reduced to Fe^2+^. Then iron-oxidizing microorganisms re-oxidize Fe^2+^ to Fe^3+^, and sulfur-oxidizing microorganisms oxidize RISCs to sulfuric acid. Consequently, the minerals get dissolved (Sand et al., 2001). The typical microorganisms involved in bioleaching are mainly from the genera *Acidithiobacillus*, *Leptospirillum*, *Acidiphilium*, *Sulfobacillus*, *Ferroplasma*, *Sulfolobus*, *Metallosphaera*, and *Acidianus* (Rawlings, 2002; Yin et al., 2020). Industrial bioleaching mostly is undertaken by percolation in heaps where the abundant microorganisms are usually *Acidithiobacillus*, *Leptospirillum*, and *Sulfobacillus* (Acosta et al., 2014; Chen et al., 2020). For heap bioleaching, each heap surface is sprayed with basal salt solution, and air is injected at the bottom to enhance the microbial growth. The leached copper is removed selectively from the pregnant leach solution (PLS) by SX and finally recovered via electrowinning (EW).

For economic purposes, the raffinate after stripping is normally returned to the heaps. However, the extractants cannot be separated quantitatively from the recycled solution. So the raffinate contains entrained (dissolved) SX chemicals which are toxic to microorganisms. The most applied copper extractants are hydroxyoxime extractants such as LIX reagents. Torma and Itzkovitch (1976) investigated the effects of six LIX reagents on the oxidation of chalcopyrite by *Acidithiobacillus ferrooxidans*. Due to a decrease in the oxygen uptake, the LIX reagents were assumed to have an effect causing an inhibition of bacterial activity, but the effect was small. Dopson et al. (2006) showed that both LIX 622 and LIX 973 cause decreased Fe(II) oxidation rates and increased lag times for *Sulfolobus metallicus* BC65 already in the presence of 0.001% (v/v) SX chemicals. Watling et al. (2009) reported that 50 mg/L SX reagent (LIX 984N 20% v/v in Shellsol 2046) did not cause significant negative effects on the growth of *At. ferrooxidans* or on its bioleaching performance. However, in the case of *Sulfobacillus thermosulfidooxidans*, copper extraction was reduced to about one-third with 50 mg/L SX reagent. Similar work has been done (Mazuelos et al., 1999; Chen et al., 2010; Davis-Belmar et al., 2012; Liu et al., 2015), but it focused only on one species, plus the type and the concentration of the SX reagent applied in these studies are not identical. Consequently, no comparable data show whether those microorganisms have different sensitivity toward one SX reagent and, if so, how high concentration of the SX reagent each species can resist. Besides, these studies evaluated only the impact of SX reagents on the growth of planktonic microorganisms, whereas biofilms were neglected.

Biofilms are defined as aggregates of microorganisms embedded in a self-produced matrix of extracellular polymeric substances (EPS). The latter consists of a conglomeration of different types of biopolymers such as polysaccharides, proteins, nucleic acids, and lipids (Flemming and Wingender, 2010; Flemming et al., 2016). EPS help microorganisms to firmly adhere on a surface and provide structural support for the biofilm. Thus, it is vital for biofilm development (Stoodley et al., 2002). Biofilms are a form of collective life with emergent properties that confer many advantages on their inhabitants, allowing them to survive under very harsh conditions such as drying or nutrient deprivation (Flemming et al., 2021). Except in the oceans, biofilms dominate in all habitats on the surface of the Earth, and, overall, 40–80% of cells on Earth reside in biofilms (Flemming and Wuertz, 2019). Formation of biofilms is also general for leaching microorganisms, and it is reported that biofilms initiate and enhance bioleaching (Crundwell, 1996; Bond et al., 2000; Bellenberg et al., 2015). In heap bioleaching the surfaces of the mineral piles are wetted by spraying, and the liquid flows through the heap and is collected at the bottom and recycled. Obviously, formation of biofilm is key for the microorganisms to survive and be metabolically active. However, information on how SX reagents affect biofilm development and its metabolism are missing. Another issue is that heap leaching is usually applied to extract copper from low-grade chalcopyrite waste rock, while most of the reported work was done with chalcopyrite concentrates. It is still unknown what the impact of the SX reagents is on the microorganisms growing on low-grade chalcopyrite waste rock.

In this work, the effect of SX reagent (LIX984N in kerosene) on copper extraction from waste rock by three typical heap bioleaching strains, *At. ferrooxidans*, *Leptospirillum ferrooxidans*, and *Sb. thermosulfidooxidans*, was studied. The bacteria were first cultivated in the presence of the SX reagent with Fe^2+^ or S° as sole energy source, from which the highest concentration of the SX reagent for the bacterial survival was determined. The results also clearly show that the three species have different sensitivity to the SX reagent. Then with tolerable concentrations of the SX reagent, bioleaching of chalcopyrite waste rock by the three species was tested. The inhibitory effect of the SX reagent on bioleaching was exerted, and we propose that the inhibition might be related to an interference/change of bacterial metabolism. The bacteria applied for bioleaching were not previously adapted to the SX reagent, but our study provides a comparable and detailed information on the impacts of the SX reagent on bioleaching microorganisms, which more or less reflects the events in a newly built heap. However, in the available literature such data are not given. Our study also helps to better understand the microorganisms in heaps and to establish strategies to maintain high bacterial activity and heap leaching rates.

## Materials and Methods

### Strains and Cultivation

*Acidithiobacillus ferrooxidans* ATCC 53993 was originally purchased from American Type Culture Collection (ATCC). *L. ferrooxidans* DSM 2705 and *Sb. thermosulfidooxidans* DSM 9293 were purchased from Deutsche Sammlung von Mikroorganismen und Zellkulturen (DSMZ), Germany. The strains were cultivated in Mackintosh basal salt medium (MAC) (Mackintosh, 1978). FeSO_4_⋅7H_2_O and elemental sulfur powder (Carl Roth, Germany) were used as energy source. *L. ferrooxidans* and *At. ferrooxidans* were incubated with 4 g/L Fe^2+^ at 28°C, while *Sb. thermosulfidooxidans* was incubated in the presence of 0.02% yeast extract with 4 g/L Fe^2+^ or 1% S° at 45°C. For cultivation, the initial pH was 1.8 with Fe^2+^ as energy source or 2.5 with S° as energy source. All strains were incubated on a rotary shaker at 120 rpm.

### Preparation of Chalcopyrite Waste Rock Slices and Grains

Chalcopyrite waste rock was collected from Dexing Copper Mine, Jiangxi Province, China. It contained 0.1% Cu, 12.9% Fe, 1.8% S, 58.1% SiO_2_, 13.8% Al_2_O_3_, and 1.8% CaO. For preparation of slices, the chalcopyrite waste rocks were first cut into 3-mm-thick slides. Afterward each slice was polished successively with water on 500-, 800-, and 1,200-grit silicon carbide grinding papers (Struers, Germany). To obtain grains, the chalcopyrite waste rocks were crushed with a jaw crusher (BB 1/A, Retsch, Germany) and then ground with a disc-swing mill (HSM 100M, Herzog). After wet sieving (Test sieves, Retsch, Germany), grains with a size of 50–200 μm were used in this study.

For cleaning and sterilization, both chalcopyrite waste rock slices and grains were washed firstly in three volumes of 0.1 M EDTA in 0.4 M NaOH for 10 min under stirring, followed by three iterations of washing with one volume of acetone. Finally, they were dried at 60°C overnight and then sterilized at 120°C for 10 h under a nitrogen atmosphere in a drying cupboard.

### The Solvent Extraction Reagent

The SX reagent used in this study was LIX984N at a concentration of 2.5% v/v. Commercial-grade sulfonated kerosene was used as diluent. LIX984N, a mixture of 2-hydroxy-5-non-ylacetophenone oxime and 5-non-ylsalicylaldoxime, was purchased from BASF Greater China. Hamilton Microliter syringes were used to add the SX reagent into the microbial culture medium. The syringes were washed with ultrapure water and acetone before and after the injection.

### Impact of the Solvent Extraction Reagent on Bioleaching and Biofilm Formation

For bioleaching tests all strains were at first adapted to the chalcopyrite waste rock by incubating them with 1% chalcopyrite waste rock supplemented with FeSO_4_⋅7H_2_O. The amount of Fe^2+^ was gradually reduced till the bacteria could grow only with chalcopyrite waste rock.

Bioleaching experiments in the presence of the SX reagent were carried out in narrow-neck Erlenmeyer flasks at 120 rpm for at least 21 days with a pulp density of 2% (w/v) and an initial pH of 1.9. *At. ferrooxidans* and *L. ferrooxidans* were incubated at 28°C, while *Sb. thermosulfidooxidans* was incubated with 0.02% yeast extract at 45°C. Planktonic cell number, pH, and dissolved copper were monitored.

To follow biofilm development in the presence of the SX reagent, a cell suspension with a density of 1 × 10^8^ cells/mL was incubated at 80 rpm in wide-neck Erlenmeyer flasks containing SX reagent and chalcopyrite waste rock slices. A tweezer was used to manipulate the slices. After washing with fresh MAC medium and sterilized ultrapure water, they were used for staining.

All experiments were performed in triplicate.

### Determination of Cell Count, pH, Iron, Copper, and Sulfate

The planktonic cell counts were determined using a Thoma counting chamber (Assistent, Germany) and a light microscope (Leica DMLS, Wetzlar GmbH) in phase contrast mode with × 400 magnification.

The pH was determined with a digital pH meter (Model pH 537, WTW, inLab^®^ 422 Combination Semi-micro pH Electrode, Mettler Toledo).

The concentration of Fe^3+^ was quantified by the following procedure described by Tamura (Tamura et al., 1974). The principle is that ferrous ions and 1,10-phenanthroline form red colored complexes, which can be measured spectrophotometrically at 492 nm. After measuring the concentration of ferrous ions, the total iron can be quantified by adding hydroxylamine which reduces ferric ions to ferrous ions. The concentration of total iron minus the concentration of ferrous ions is then equal to the concentration of ferric ions. The samples were measured in triplicate within microtiter plates with a UV-Vis spectrophotometer (TECAN, Infinite M200 pro) equipped with the software Tecan i-control.

Copper ions were determined by flame atom absorption spectrometry at a wavelength of 324.8 nm (Perkin Elmer 1100B) according to the German standard methods for the examination of water, waste water, and sludge; cations; determination of copper was by atomic absorption spectrometry (AAS) (DIN 38406-7).

The concentration of sulfate was determined by ion exchange chromatography (IC). A Dionex DX-500 system in combination with an eluent generator (EG50), a conductivity detector (CD20), and an autosampler (AS3500) was used. The system was controlled using the chromatographic software Chromeleon Version 6.7. An analytical column with anion exchange resin as stationary phase (IonPac AS17, 2 × 250 mm, Dionex, United States) and a guard column (IonPac AG17, 50 mm, Dionex, United States) were used. A flow rate of 0.25 mL/min and a suppressor current of 50 mA were applied for the measurement. The injection volume of the autosampler was 10 μL. Potassium hydroxide was used as eluent. The gradient system was as follows: 0–2.5 min 10 mM KOH; 2.5–3.5 min 20 mM KOH; 3.5–4.5 min 30 mM KOH; 4.5–5.5 min 40 mM KOH; 5.5–6.5 min 50 mM KOH; and 6.5–8.5 min 10 mM KOH). Samples were diluted 1:10 with 5 mM phosphate buffer (40% 50 mM KH_2_PO_4_, 60% 50 mM K_2_HPO_4_, pH 7) to avoid (metal) precipitation in the column and incubated for 30 min. Afterward samples were centrifuged (Biofuge, Heraeus Sepatech) for 10 min at 8,000 rpm. The supernatants were diluted further 1:10 with bidistilled water and then measured. Samples were measured in duplicate.

### Most Probable Number Determination

Most probable number (MPN) tests, according to Escobar and Godoy (1999), were carried out in 96 deep-well plates (2 mL) to determine the number of active Fe(II) oxidizers in the bioleaching system. Each well was filled with sterile 900 μL MAC medium containing 4 g/L Fe^2+^. For the MPN determination, 1 mL culture was first transferred to a sterile test tube with 9 mL MAC medium and vortexed. Then 100 μL of this dilution was added to a well of the plate. After mixing by a pipette, 100 μL inoculum was transferred to the next well. This step was repeated several times. The culture was diluted 8 times in total. The inoculated plates were then incubated statically. Each determination was performed in triplicate.

### Microcalorimetry

Metabolic activities of microorganisms under the influence of SX reagents were evaluated with a multi-channel microcalorimeter (TAM III, TA Instruments) by recording metabolic heat flow while bioleaching. The microcalorimetric measurement is based on the isothermal ampoule method. On the 2nd, 4th, 7th, 10th, 14th, 17th, and 21st day of bioleaching the heat flow was measured, respectively. For each set of experiment, bacteria were inoculated into seven identical flasks containing SX reagents and 2% (w/v) chalcopyrite waste grains in 50 mL MAC at pH 1.9. Then they were incubated on a rotary shaker at 120 rpm. One flask was taken out for each measurement. After determination of cell concentration and pH, the inoculum together with chalcopyrite waste was filtered through a sterile filter with a pore size of 0.22 μm. Then the chalcopyrite waste rock was collected, weighted, and transferred into two disposable ampoules. The ampoules were sealed and introduced into the microcalorimeter, allowing temperature equilibration for 15 min before running the measurement. The chalcopyrite waste rock was collected afterward and dried at 110°C overnight to calculate their water content. Values of the heat flow are expressed as microwatt per gram of dry chalcopyrite waste (μW/g). All experiments were carried out within 24 h and at least in triplicate.

### Atomic Force Microscopy and Epifluorescence Microscopy Instrumentation and Performance

A NanoWizard II atomic force microscope (JPK Instruments, Berlin, Germany) combined with an upright epifluorescence microscope (Axio Imager A1m, Zeiss, Germany) via a BioMaterial workstation (JPK Instruments) was used to locate and scan cells or biofilms. The BioMaterial workstation allows a sample transfer between the atomic force microscope and the epifluorescence microscope, giving the same position for both microscopes. Usage of both microscopes can ensure the scanned objects are cells.

For epifluorescence microscopy (EFM) imaging, the biofilms were stained with 6 μM SYTO9 (Invitrogen, Germany) and 50 μg/mL fluorescently labeled lectin Concanavalin A (Con A) (EY Laboratories). SYTO 9, showing as a green signal, was used to stain the cells for their location within the biofilm. Con A, showing as a red signal, was used to visualize the distribution of EPS. All EFM images in this study show the combination signals of Con A and SYTO 9 (indicated as light yellow, i.e., the combination of red and green). For atomic force microscopy (AFM) imaging, a CSC38/NO AL (Mikromasch, Tallinn, Estonia) probe was used, and cantilever B with the following parameters was chosen: length, 350 μm; width, 32.5 μm; thickness, 1.0 μm; resonance frequency, 10 kHz; shape, cone with a full cone angle of 40°; and force constant, 0.03 N/m. Contact mode with a set point below 1 nN was applied, and the scan rate ranged from 0.1 to 0.5 Hz. To obtain EFM and AFM images of the same location, biofilms were at first observed under the epifluorescence microscope and then transferred to the atomic force microscope for scanning.

### Statistical Analysis

One-way analysis of variance (ANOVA) followed by the least significant difference (LSD) test was carried out using IBM SPSS Statistics 22 software to determine differences in cell growth; pH; and concentrations of Fe^3+^, SO_4_^2–^, and Cu^2+^ in the presence of the SX reagent.

## Results and Discussion

### Impact of the Solvent Extraction Reagent on Bacterial Growth

**FIGURE 1:**
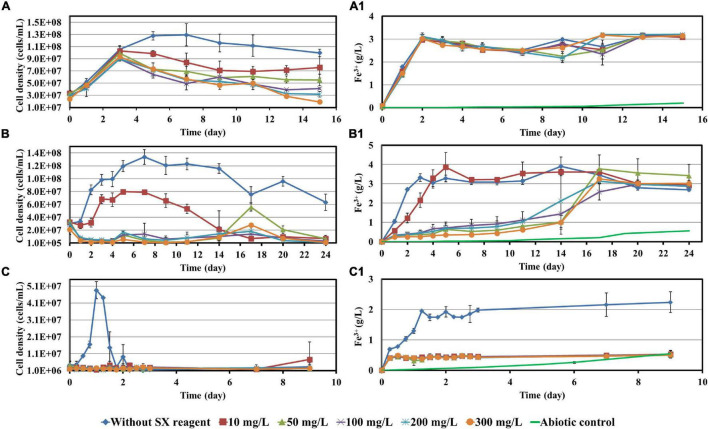
Impact of the SX reagent on growth and Fe^2+^ oxidation by *At. ferrooxidans* **(A,A1)**, *L. ferrooxidans* **(B,B1)**, and *Sb. thermosulfidooxidans* **(C,C1)** with Fe^2+^ as energy source.

**FIGURE 2:**
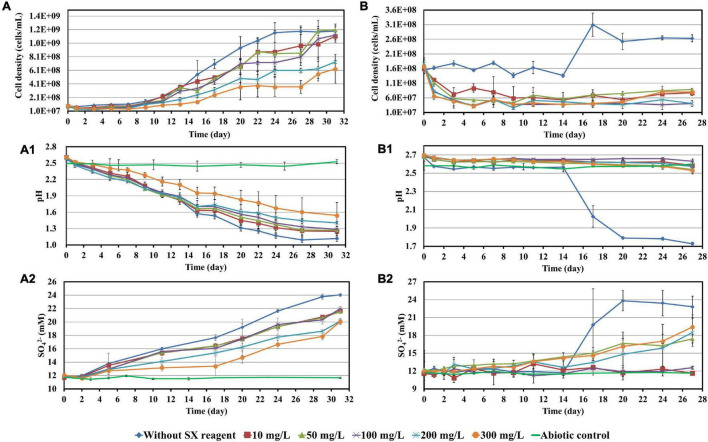
Impact of the SX reagent on growth and S° oxidation by *At. ferrooxidans* **(A–A2)** and *Sb. thermosulfidooxidans* **(B–B2)** with elemental sulfur powder as energy source.

Before bioleaching of chalcopyrite waste rock, the impact of 10, 50, 100, 200, and 300 mg/L SX reagent on the growth of *At. ferrooxidans*, *L. ferrooxidans*, or *Sb. thermosulfidooxidans* with Fe^2+^ or S° as sole energy source was checked. Parameters such as cell density, pH, and concentrations of Fe^3+^ and SO_4_^2–^ were recorded and are shown in Figures 1, 2.

When *At. ferrooxidans* was grown on Fe^2+^, the cells in all assays entered the log phase with the same growth rate (the slope) once they were inoculated (Figure 1A). After 5 days without the SX reagent the cells entered the stationary phase. With 10 and 50 mg/L SX reagent, after the increase in the first 3 days, the cell density started to decrease and then leveled off from the 9th day on. With 100 and 200 mg/L SX reagent, the cell density also started to decrease from the 3rd day on and then leveled off from the 13th day on. With 300 mg/L SX reagent, the number decreased from the 3rd day on till the end. At the end of the experiment the cell density in all assays with the SX reagent was significantly lower than that without (*p* < 0.05), indicating the SX reagent accelerated the death of the cells. Inhibition of Fe(II) oxidation was not detected, because the Fe(III)-generating rate (the slope) was identical in all assays (Figure 1A1). The results are consistent with those reported by Watling et al. (2009), in which with Fe^2+^ as energy source 50 mg/L SX reagent (LIX 984N 20% v/v in Shellsol 2046) had little impact on *At. ferrooxidans* in terms of Fe^2+^ oxidation rate and cell growth.

However, if *At. ferrooxidans* was grown on S°, two-stage cell growth and a delay in sulfur oxidation were detected (Figure 2A), indicating a negative effect of the SX reagent. Specifically, the cell number in all assays increased from the 7th day on. Without the SX reagent (control) it leveled off at 1.2 × 10^9^ cells/mL from the 24th day on, while with the SX reagent it increased in two stages. At the end of the experiment the cell numbers in the assays with 10, 50, and 100 mg/L SX reagent almost equaled those without, while with 200 and 300 mg/L SX reagent they were significantly lower (*p* < 0.05). The decreased pH (Figure 2A1) and the increased sulfate (Figure 2A2) indicate some bio-oxidation of S°, but it was inhibited in the presence of the SX reagent. In the end the concentrations of SO_4_^2–^ in all assays with the SX reagent were significantly lower than in the control (*p* < 0.05). Watling et al. (2012) tested the effect of LIX984N (20% v/v in Shellsol 2046) on tetrathionate oxidation by *Acidithiobacillus caldus*. According to their results, LIX984N prolonged the bacterial lag time, and higher concentrations caused longer delays before tetrathionate oxidation commenced, which is different from our results. A very long lag phase in this study (7 days versus 10 h) might be responsible for the difference, since it gave some bacteria a chance to adapt to the SX reagent and they could grow fast just after the lag phase. According to Figure 2A, the higher the concentration of the SX reagent was, the less cells could adapt. For the rest of the cells, prolonged time was needed for adaptation. Consequently, the cell growth occurred in two phases.

*Leptospirillum ferrooxidans* can oxidize Fe^2+^ only. With the SX reagent the bacteria showed a typical delay in growth (Figure 1B) and Fe^2+^ oxidation (Figure 1B1). The duration of the delay was negatively related to the concentration of the SX reagent. A low cell density and Fe^3+^ concentration indicate that the SX reagent inhibited the bacterial growth and Fe^2+^ oxidation. Mazuelos et al. (1999) also showed that for a *L. ferrooxidans*-containing mixed culture, if Fe^2+^ was given as energy source, a rising concentration of the SX reagent (15% LIX 64 in kerosene) caused a decrease in the Fe^2+^ oxidation rate and an increase in the lag phase.

Our results show that *Sb. thermosulfidooxidans* is quite sensitive to the SX reagent, if it is grown on Fe^2+^. In the absence of the SX reagent it grew fast, and Fe^2+^ was oxidized completely within 1 day. However, in the presence of the SX reagent neither cell growth nor Fe^2+^ oxidation was detected (Figures 1C,C1). which is consistent with the results reported by Watling et al. (2009). An increased incubation time allows the bacteria to adapt to the SX reagent. Collinson et al. (2011) found that more than 1 year was required to achieve habituation to 2 mg/L 4-non-ylphenol, the most inhibitory component of LIX984N, when *Sb. thermosulfidooxidans* was grown on Fe^2+^.

Similar results were obtained when *Sb. thermosulfidooxidans* was grown on S°. In the absence of the SX reagent its growth was quite well, while in the presence of the SX reagent no growth was observed (Figure 2B). However, in the assays with 50, 200, or 300 mg/L SX reagent, the pH decreased (Figure 2B1), and the concentrations of sulfate increased (Figure 2B2) from the 14th day on, indicating a commencement of bio-oxidation of S°. No directly reported comparable data have been found.

According to the results described above, some points can be summarized:

1. When Fe^2+^ was available for growth, the SX reagent showed small negative effects on the growth and Fe^2+^ oxidation of *At. ferrooxidans*. However, when Fe^2+^ was depleted, the presence of the SX reagent accelerated the death of the cells. When S° was available, the effects of inhibition on the bacterial growth and S° oxidation were observed. The presence of the SX reagent made the cell numbers increase in two stages, which might be related with the differences in bacterial adaptation.

2. The presence of the SX reagent delayed the growth and oxidation of *L. ferrooxidans*.

3. When *Sb. thermosulfidooxidans* was grown on Fe^2+^, the SX reagent inhibited bacterial growth and Fe^2+^ oxidation completely. An oxidation of S° by the bacteria in the presence of increased concentrations of the SX reagent seemed to occur.

### Impact of the Solvent Extraction Reagent on Bioleaching of Chalcopyrite Waste Rock by *Acidithiobacillus ferrooxidans*

**FIGURE 3:**
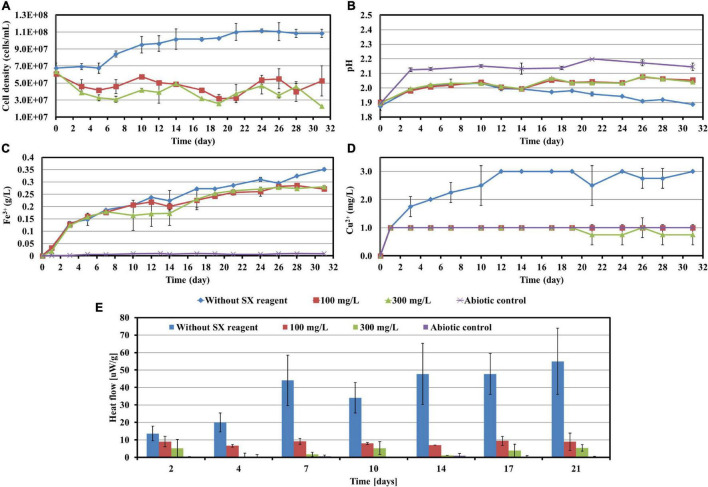
Impact of the SX reagent on bioleaching of chalcopyrite waste rock by *At. ferrooxidans*. Changes of cell density **(A)**, pH **(B)**, Fe^3+^ **(C)**, Cu^2+^ **(D)**, and heat flow of the filtered chalcopyrite waste rock **(E)**, were recorded during bioleaching.

To investigate the impact of the SX reagent on bioleaching of chalcopyrite waste rock by *At. ferrooxidans*, concentrations of 100 and 300 mg/L of the SX reagent were chosen, since the results in section “Impact of the Solvent Extraction Reagent on Bacterial Growth” show that the inhibitory effects were small. In the absence of the SX reagent (control), the final cell density of the bacteria reached 1.1 × 10^8^ cells/mL (Figure 3A). In the presence of the SX reagent the cell density fluctuated in a range from 3.0 × 10^7^ to 6.0 × 10^7^ cells/mL. The pH of the leaching system increased dramatically at the beginning, which can be attributed to the acid-consuming components in the chalcopyrite waste. It did not decline till the end of the experiments, except for the assays without the SX reagent (Figure 3B). At the end, the pH in the controls was significantly lower than that in the experimental assays (*p* < 0.05). On the Fe(II) oxidation the SX reagent did not exert a negative effect (Figure 3C) because no statistically significant differences were detectable between the control and the experimental sets (*p* > 0.05). However, a significantly reduced concentration of Cu^2+^ (*p* < 0.05) was detected, indicating that copper extraction became inhibited seriously (Figure 3D). Without the SX reagent the final concentration of Cu^2+^ was 3 mg/L (15% of total copper), while with the SX reagent the Cu^2+^ was only released by chemical leaching to 1 mg/L (5% of total copper). Although it has been reported that the SX reagent (20% v/v LIX984N in Shellsol 2046) has only little inhibitory effect on copper extraction by *At. ferrooxidans* (Watling et al., 2009), that study had focused on copper extraction from chalcopyrite concentrates rather than chalcopyrite waste rock. Our results clearly show that the SX reagent inhibited *At. ferrooxidans* from extracting copper from chalcopyrite waste rock. A possible explanation is that *At. ferrooxidans* did not oxidize RISCs, which caused passive layers to form on the chalcopyrite waste rock and gradually interfered with the bioleaching. Some hints can also be found. Without the SX reagent, the decrease in pH started from the 10th day, when the RISCs should have been formed. Coincidentally with the SX reagent the increase in Fe^3+^ became slow from around the 10th day on, and from the 19th day on the concentration of Fe^3+^ almost stabilized at 0.26 g/L. The inhibition might not occur for chalcopyrite concentrates because they provide much more Fe^2+^ than that in the same amount of the chalcopyrite waste rock, and the oxidation of Fe^2+^ by *At. ferrooxidans* is not influenced. Consequently, large amounts of Fe^3+^ will be released, and these Fe^3+^ can oxidize RISCs (Sand et al., 2001). Similar inhibitory effects of the SX reagent on bioleaching of chalcopyrite waste rock by *At. ferrooxidans* have been reported by Liu et al. (2015). In their study, the impact of entrained and dissolved LIX984N on bioleaching of chalcopyrite waste rock by *At. ferrooxidans* was checked. After contact with different concentrations of the SX reagent (2.5% LIX984N in kerosene), the medium was separated and bioleaching was applied. Even though the SX reagent had been removed, some inhibitory effects on bioleaching were still evident. In the control, 26% of the total copper was extracted, whereas 21% of the copper was extracted if the medium had been in contact with the SX reagent. The authors pointed out that the decreased copper extraction might be related with a change/breakage of the bacterial cells, because cell walls and cytoplasmic membranes became distorted and intracellular vacuoles increased in number and volume. However, they did not show any data on pH and sulfate concentrations, but combined with our results it can be deduced that the change/breakage of the bacterial cells may cause *At. ferrooxidans* to be unable to oxidize RISCs, which is negatively affecting bioleaching.

The results of the calorimetric measurements are illustrated in Figure 3E. Isothermal microcalorimetry is a sensitive method that can measure heat production rates of less than a microwatt. Thus, it can be used to monitor metabolism and growth of a relatively small number of microbial cells at any chosen temperature (Braissant et al., 2010). Isothermal microcalorimetry has been successfully applied for monitoring of bioleaching (Sand et al., 1993; Schippers et al., 1995; Hedrich et al., 2018). It is also an excellent tool for investigations on inhibition of cell growth. Sand et al. (2007) recorded heat production of *At. ferrooxidans*-like bacteria during bioleaching of pyritic mine waste over a period of 3 years. They found that bioleaching becomes inhibited if sodium dodecyl sulfate (SDS) had been supplemented. According to their results, without SDS the heat produced by the bacteria amounted to 55 μW/g waste rock (indicating a high bioleaching activity) and decreased to 10 μW/g waste rock or below with SDS addition (indicating a moderate to low bioleaching activity). Our data are similar. In our study without the SX reagent the bioleaching activity of *At. ferrooxidans* increased from 13 μW/g chalcopyrite waste rock to 54 μW/g, whereas with the SX reagent the bioleaching activity decreased to 8 μW/g in the presence of 100 mg/L SX reagent or to 3 μW/g in the presence of 300 mg/L SX reagent. These data are a further proof of the inhibitory effect of the SX reagent on bioleaching of chalcopyrite waste rock by *At. ferrooxidans*.

Most probable number data indicating living cells are shown in Table 1. Since for the MPN determination Fe^2+^ was used as substrate, the data are relative viability numbers of Fe(II)-oxidizing cells of *At. ferrooxidans* in the bioleaching system. Without the SX reagent the number of active Fe(II)-oxidizing cells increased within 1 week to more than 1.1 × 10^8^ cells/mL, whereas in the assays with 100 and 300 mg/L SX reagent it took 2 to 3 weeks to achieve that number, respectively. The increased cell numbers in the assays with the SX reagent demonstrate that the bacteria remained alive till the end of the experiment and were able to adapt to the SX reagent. The higher the concentration of the SX reagent was, the longer was the adaptation time. The results also indicate that primarily the bio-oxidization of RISCs became inhibited, because almost all cells were detected as iron oxidizers.

**TABLE 1:** Cell numbers of active Fe(II)-oxidizing *At. ferrooxidans* determined by the most probable number method during bioleaching of chalcopyrite waste rock with or without the SX reagent.

Time (days)	Without SX reagent (cells/mL)	100 mg/L (cells/mL)	300 mg/L (cells/mL)
1	(4.5 ± 0.2) × 10^7^	(4.3 ± 0.5) × 10^7^	(2.7 ± 1.7) × 10^7^
7	>(1.1 ± 0.0) × 10^8^	(3.2 ± 1.6) × 10^7^	(1.8 ± 0.4) × 10^7^
14	>(1.1 ± 0.0) × 10^8^	>(1.1 ± 0.0) × 10^8^	(0.9 ± 0.0) × 10^7^
21	>(1.1 ± 0.0) × 10^8^	>(1.1 ± 0.0) × 10^8^	>(1.1 ± 0.0) × 10^8^

**FIGURE 4:**
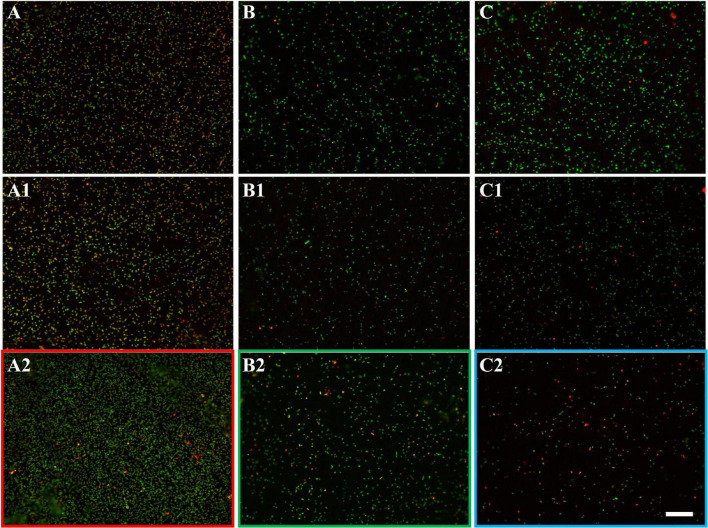
EFM images of biofilms developed without **(A series)**, with 100 mg/L **(B series)**, or with 300 mg/L SX reagent **(C series)** on chalcopyrite waste rock by *At. ferrooxidans*. Images **(A–C)** show the 1-day-old biofilms/attached cells; images **(A1–C1)** show 3-day-old biofilms/attached cells; and images **(A2–C2)** show 7-day-old biofilms/attached cells. The scale bar is 20 μm, and all images share the scale bar.

**FIGURE 5:**
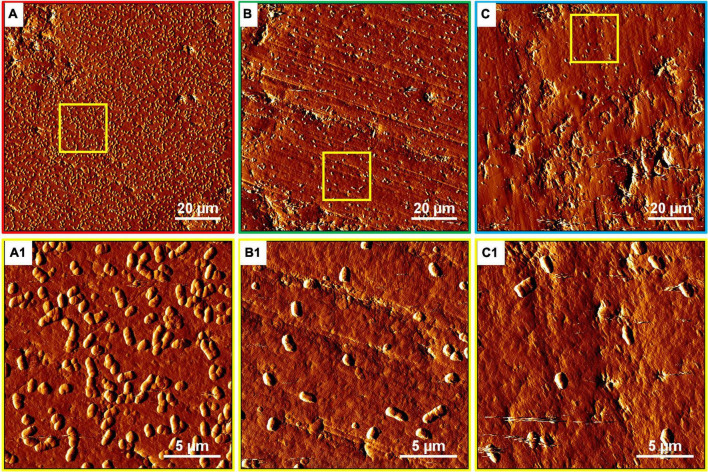
AFM images of biofilms developed without **(A series)**, with 100 mg/L **(B series)**, or with 300 mg/L SX reagent **(C series)** on chalcopyrite waste rock by *At. ferrooxidans*. The areas shown in images **(A–C)** are in accordance with Figures 4A2,B2,C2, respectively. Images **(A1–C1)** show the areas framed in images **(A–C)**, respectively.

The development of biofilms on chalcopyrite waste rock by *At. ferrooxidans* within 7 days is shown in Figures 4, 5, which are EFM and AFM images. Figures 4A2–C2 are in accordance with Figures 5A–C, respectively. The corresponding images are framed in the same color. The EFM images provide information on distribution of attached cells/biofilms, and AFM images show the morphology and architecture of the cells/biofilms. Figures 5A1–C1 are zoom pictures of the areas framed in Figures 5A–C, respectively. After 1 day of incubation, the numbers of attached cells in the assays with the SX reagent were equal to those without, as indicated by similar fluorescent signals (Figures 4A–C). Later on in the assays without the SX reagent, the fluorescent signals got enhanced (Figures 4A1,A2) because of the densely covered colonies (Figures 5A,A1). This shows that biofilms are formed. In the presence of the SX reagent, the bacteria seem not to develop biofilms. To be precise, with 100 mg/L SX reagent, the fluorescent signals barely changed (Figures 4B1,B2), and only single cells occurred (Figures 5B,B1). Considering the low heat production in the first 7 days additionally (Figure 3E), one can conclude that those single cells were attached cells and biofilms had not yet developed. With 300 mg/L SX reagent, the fluorescent signals gradually got weak (Figures 4C1,C2) and large areas were exposed, indicating that the initially attached cells gradually detached. Only some single cells were visible (Figures 5C,C1), and these produced very little low heat (Figure 3E). The results demonstrate that 300 mg/L SX reagent inhibited *At. ferrooxidans* preventing the cells from forming biofilms on chalcopyrite waste rock during the first 7 days. Attached cells/biofilms play an important role during bioleaching. It is reported that within the first 4–5 days only the biofilm subpopulation is responsible for metal sulfide dissolution (Bellenberg et al., 2015). Gautier et al. (2008) found that if cells of *S. metallicus* were prevented from reaching chalcopyrite, the cell population would not grow. Consequently, according to their data, during copper dissolution, only 50% of the amount was reached if contact was allowed. That value was comparable to the aerated abiotic control. Furthermore, these authors pointed out that an oxidation of chalcopyrite by ferric ions would lead to a formation of sulfur-containing passivating layers, and the attached cells of *S. metallicus* preferentially catalyzed the oxidation of such sulfur compounds. Thus, the attached cells dissolved the passivating layers and enhanced the oxidative action of the ferric ions on chalcopyrite. Back to our study, the SX reagent showed negative effects on biofilm formation on chalcopyrite waste rock by *At. ferrooxidans*. Consequently, there was not enough energy source to support the bacterial growth, and later the passivating layers could not be dissolved in time, thereby weakening the bioleaching.

Based on the results above, it can be concluded that 100 and 300 mg/L SX reagent can hardly inhibit *At. ferrooxidans* from oxidizing Fe^2+^ but seriously interferes with the biofilm formation and the oxidization of RISCs. Consequently, sulfur-containing passivating layers gradually can hinder the bioleaching of chalcopyrite waste rock.

### Impact of the Solvent Extraction Reagent on Bioleaching of Chalcopyrite Waste Rock by *Leptospirillum ferrooxidans*

**FIGURE 6:**
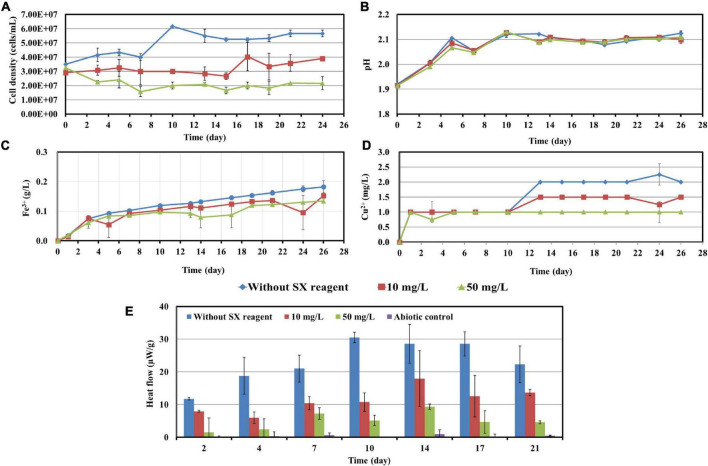
Impact of the SX reagent on bioleaching of chalcopyrite waste rock by *L. ferrooxidans*. Changes of cell density **(A)**, pH **(B)**, Fe^3+^ **(C)**, Cu^2+^ **(D)**, and heat flow of the filtered chalcopyrite waste rock **(E)**, were recorded during bioleaching.

Cells of *L. ferrooxidans*, unlike *At. ferrooxidans*, are sensitive to the SX reagent. Thus, concentrations of 10 and 50 mg/L SX reagent were applied for the bioleaching tests and the data were recorded in Figure 6. *L. ferrooxidans* can oxidize Fe^2+^ only, so its growth on chalcopyrite waste rock, as expected, was not as good as that of *At. ferrooxidans*.

**FIGURE 7:**
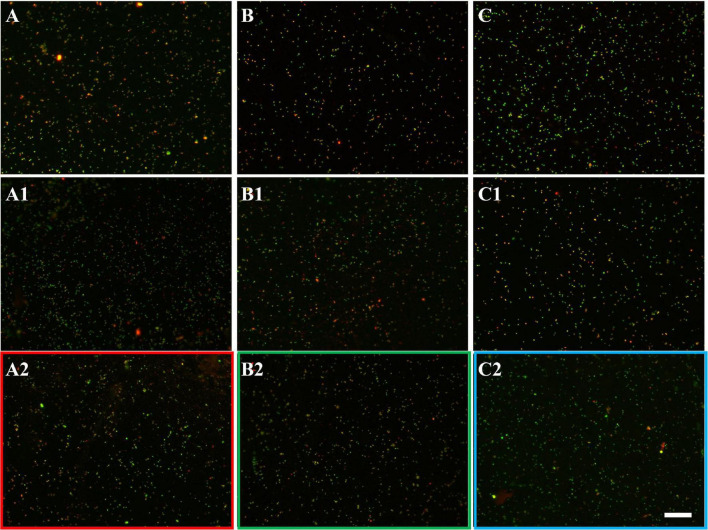
EFM images of biofilms developed without **(A series)**, with 10 mg/L **(B series)**, and with 50 mg/L SX reagent **(C series)** on chalcopyrite waste rock by *L. ferrooxidans*. Images **(A–C)** show 1-day-old biofilms/attached cells; images **(A1–C1)** show 3-day-old biofilms/attached cells; and images **(A2–C2)** show 7-day-old biofilms/attached cells. The scale bar is 20 μm, and all images share the scale bar.

**FIGURE 8:**
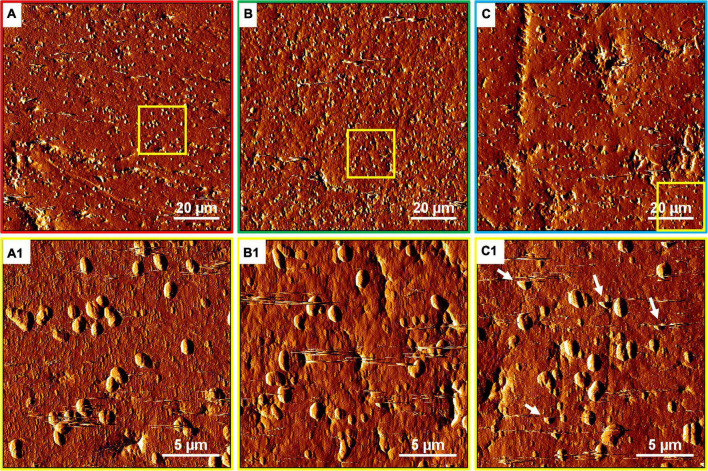
AFM images of biofilms developed without **(A series)**, with 10 mg/L **(B series)**, and with 50 mg/L SX reagent **(C series)** on chalcopyrite waste rock by *L. ferrooxidans*. The areas shown in images **(A–C)** are in accordance with that in Figures 7A2,B2,C2, respectively. Images **(A1–C1)** show the areas framed in images **(A–C)**, respectively. The arrows indicate detached cells during scanning.

In the absence of the SX reagent (control), the maximum cell density only amounted to 6 × 10^7^ cells/mL (Figure 6A). The numbers of active cells increased to 2.9 × 10^7^ cells/mL after 7 days of incubation but afterward decreased as bioleaching proceeded (Table 2). Although the fluorescent signals hardly changed in the first 7 days (Figures 7A–A2), accumulation of cells was observed (Figures 8A,A1), and an increased heat production was detected (Figure 6E), indicating the formation of biofilms and the performance of bioleaching. From the 10th day on, the heat production leveled off at around 30 μW/g chalcopyrite waste rock and then decreased to 22 μW/g chalcopyrite waste rock, indicating that bioleaching became gradually hindered. The numbers of active cells started to decrease from the 10th day onward. The concentration of Fe^3+^ ions increased slowly. The amount of dissolved Cu^2+^ was 2 mg/L (10% of the total copper) at the end of the experiments.

**TABLE 2:** Cell numbers of active Fe(II)-oxidizing *L. ferrooxidans* determined by the most probable number method during bioleaching of chalcopyrite waste rock with or without the SX reagent.

Time (days)	Without SX reagent (cells/mL)	10 mg/L (cells/mL)	50 mg/L (cells/mL)
1	(4.2 ± 3.0) × 10^6^	(3.2 ± 1.6) × 10^6^	(2.2 ± 0.1) × 10^6^
7	(2.9 ± 2.0) × 10^7^	(3.2 ± 1.6) × 10^6^	(1.8 ± 0.4) × 10^5^
14	(2.6 ± 2.4) × 10^6^	(2.3 ± 0.0) × 10^5^	(2.4 ± 2.0) × 10^4^
21	(3.3 ± 1.4) × 10^5^	(4.9 ± 3.7) × 10^4^	(8.4 ± 1.3) × 10^3^

With 10 mg/L SX reagent the lag time of *L. ferrooxidans* increased to 15 days (Figure 6A). The cell density finally stabilized at 4 × 10^7^ cells/mL on the 17th day. The number of active cells in the first 7 days was 3.2 × 10^6^ cell/mL. Later on, the numbers started to decrease (Table 2). The EFM images (Figures 7B–B2) show the fluorescent signals are similar with those of the control. However, the AFM images (Figures 8B,B1) show that with 10 mg/L SX reagent the biofilms were not as robust as that in the control, because less accumulation of cells could be noticeable, and their metabolic heat production reached only half of that in the control (Figure 6E). Consequently, at the end of the experiments, only 7.5% of the total copper was extracted (Figure 6D).

With 50 mg/L SX reagent, no growth was observed (Figure 6A), and the number of active cells decreased right from the start (Table 2). The EFM images (Figures 7C–C2) show that cells attached on the surfaces of the chalcopyrite waste rock, but the procedure of the AFM imaging seems to indicate that they were inactive, because they were not tightly attached (Figures 8C,C1). The bioleaching activity was very low with an average heat production of 5 μW/g chalcopyrite waste. In the end 5% of the total copper was dissolved, equal to the value of the abiotic control. The results demonstrate that 10 mg/L SX reagent negatively affects the bioleaching of chalcopyrite waste rock by *L. ferrooxidans*, and 50 mg/L SX reagent practically stops bioleaching.

### Impact of the Solvent Extraction Reagent on Bioleaching of Chalcopyrite Waste Rock by *Sulfobacillus thermosulfidooxidans*

**FIGURE 9:**
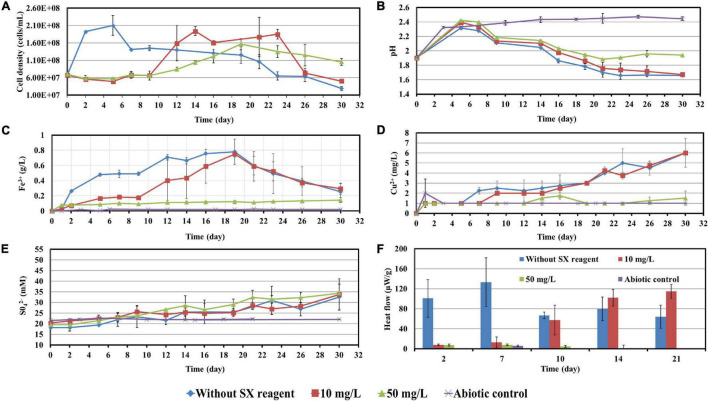
Impact of the SX reagent on bioleaching of chalcopyrite waste rock by *Sb. thermosulfidooxidans*. Changes of cell density **(A)**, pH **(B)**, Fe^3+^ **(C)**, Cu^2+^ **(D)**, SO_4_^2−^ **(E)**, and heat flow of the filtered chalcopyrite waste **(F)**, were recorded during the bioleaching.

The concentrations of the SX reagent tested with cells of *Sb. thermosulfidooxidans* were 10 and 50 mg/L due to the results from previous experiments. Surprisingly, in the presence of the SX reagent, cells of *Sb. thermosulfidooxidans* survived with the chalcopyrite waste rock (Figure 9). In addition, the bacteria exhibited differences in metabolism depending on the concentration of the SX reagent.

With 10 mg/L SX reagent the growth was inhibited during the first 9 days (Figure 9A), and almost no bioleaching occurred as indicated by the low concentration of Fe^3+^ (Figure 9C) and low heat production (Figure 9F). Afterward the cell numbers, the concentration of Fe^3+^, and the heat production increased simultaneously, which demonstrated that bioleaching had started and that the bacteria started to grow. Without the SX reagent the cell density increased rapidly to around 2 × 10^8^ cells/mL in the first 2 days and remained at that level for the next 3 days; afterward it started to decrease. With 10 mg/L SX reagent the cell density increased to 2 × 10^8^ cells/mL from the 9th day to the 14th day and then leveled off in the next 9 days. The longer stationary phase in the assays with 10 mg/L SX reagent indicated an enhanced bacterial growth, but the cell density dropped dramatically from the 23rd day on, and on the 26th day it was equal to the number for the assays without SX reagent. The MPN results (Table 3) indicated that most of the bacteria were actively Fe(II)-oxidizing cells in both assays. On the 21st day the cell density, as evaluated by MPN, decreased to 4.3 × 10^7^ cells/mL, which seems to indicate some cells had started to oxidize RISCs. From the 12th day on, the concentration of SO_4_^2–^ started to increase (Figure 9E). With 10 mg/L SX reagent, the MPN data show that on the 7th day the number of active Fe(II)-oxidizing cells was approximately 10^7^ cells/mL. On the 14th day the number had increased to more than 1.1 × 10^8^ cells/mL, and the same number was measured on the 21st day. Direct counting of samples from the 11th day till the 21st day showed that the cell density was higher than 1.1 × 10^8^ cells/mL (Figure 9A), which is in accordance with the MPN values. The results indicate that from the 11th day to the 21st day most of the cells were iron oxidizers. Because of the good growth of the actively Fe(II)-oxidizing cells, the concentration of Fe^3+^ ions in both assays increased constantly. High concentrations of Fe^3+^ ions then led to a formation of Fe precipitates, which was indicated by a sharp decline of the concentration of Fe^3+^ ions on the 19th day in Figure 9C. Bio-oxidation of RISCs also commenced, as indicated by the decrease in pH (Figure 9B) and the increase in SO_4_^2–^ ions (Figure 9E). Although 10 mg/L SX delayed the bacterial growth, the good cell growth later on made the bacteria extract the same amount of copper (30% of the total copper) as that in the assays without the SX reagent in the end (Figure 9D).

**TABLE 3:** Numbers of actively Fe(II)-oxidizing *Sb. thermosulfidooxidans* determined by the most probable number method during bioleaching with or without the SX reagent.

Time (days)	Without SX reagent (cells/mL)	10 mg/L (cells/mL)	50 mg/L (cells/mL)
1	(5.4 ± 1.5) × 10^7^	(5.7 ± 1.7) × 10^7^	(4.6 ± 0.0) × 10^7^
7	>(1.1 ± 0.0) × 10^8^	(2.3 ± 0.5) × 10^7^	(1.8 ± 0.0) × 10^3^
14	>(1.1 ± 0.0) × 10^8^	>(1.1 ± 0.0) × 10^8^	(9.3 ± 0.0) × 10^2^
21	(4.3 ± 0.5) × 10^7^	>(1.1 ± 0.0) × 10^8^	(1.9 ± 0.6) × 10^2^

With 50 mg/L SX reagent, bioleaching was inhibited completely. The concentration of Fe^3+^ did not change till the end of the experiment (Figure 9C), and an extremely low heat production was detected (Figure 9F). The MPN data show that the actively Fe(II)-oxidizing bacteria decreased, and on the 7th day their numbers had dropped to 1.8 × 10^3^ cells/mL (Table 3). On the 21st day only 190 cells/mL were detected as actively Fe(II)-oxidizing cells. As a result, the amount of dissolved copper in this assay was equal to that in the abiotic control (5% of total copper). However, the SX reagent did not fully inhibit the bacterial growth. After 9 days the cells started to multiply. The decreased pH and increased concentration of SO_4_^2–^ illustrated that they grew on RISCs which had been produced during the previous chemical leaching. After depletion of the RISCs, the cell numbers decreased and the values for pH and SO_4_^2–^ stopped changing.

**FIGURE 10:**
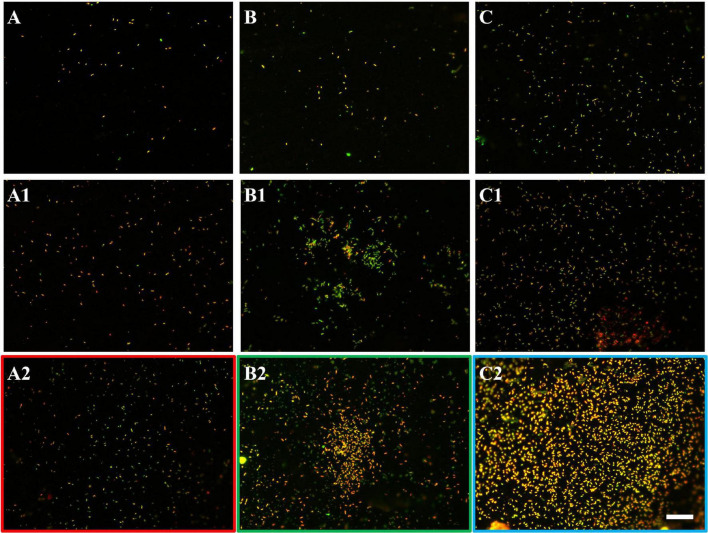
EFM images of biofilms developed without **(A series)**, with 10 mg/L **(B series)**, and with 50 mg/L SX reagent **(C series)** on chalcopyrite waste rock by *Sb. thermosulfidooxidans*. Images **(A–C)** show 1-day-old biofilms/attached cells; images **(A1–C1)** show 3-day-old biofilms/attached cells; and images **(A2–C2)** show 7-day-old biofilms/attached cells. The scale bar is 20 μm, and all images share the scale bar.

**FIGURE 11:**
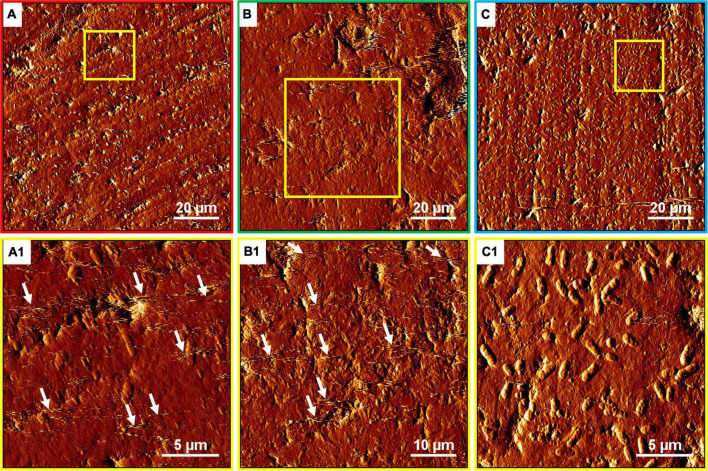
AFM images of biofilms developed without **(A series)**, with 10 mg/L **(B series)** and with 50 mg/L SX reagent **(C series)** on chalcopyrite waste rock by *Sb. thermosulfidooxidans*. Images **(A–C)** are in accordance with Figures 10A2,B2,C2, respectively. Images **(A1–C1)** show the areas framed in images **(A–C)**, respectively. The arrows indicate artifacts produced by a contaminated tip.

The EFM images in Figure 10 show that with the SX reagent *Sb. thermosulfidooxidans* formed more dense biofilms than without the SX reagent. This is indicated by the significantly strong yellow-colored fluorescent signals (Figures 10B2,C2). These signals indicate that the bacteria secreted large amounts of EPS. The AFM images (Figure 11) provide additional information. Many artifacts were produced when the samples from assays without SX reagent (Figures 11A,A1) and with 10 mg/L SX reagent (Figures 11B,B1) were scanned. Such artifacts indicate the presence of soft and sticky EPS matter. An atomic force microscope scans with a cone-shaped tip, and its resolution is decided by the radius of the tip which is usually in the range of several nanometers (Binnig et al., 1986). With such a sharp tip the soft and sticky EPS easily make contact, causing artifacts to become visible (Velegol et al., 2003). Without the SX reagent the bacteria were mostly single attached cells, and colonies were seldom observed. It is known that microorganisms prefer to attach to surface defects like cracks, holes, layers, and so on (Sand et al., 2001; Noël et al., 2010; Zhang et al., 2015). However, *Sb. thermosulfidooxidans* lacks motility. As a result, they can hardly move actively to such places and develop robust biofilms without the assistance of other microorganisms (Li et al., 2016, 2020). Thus, the randomly attached cells might detach after a while because the location is not suitable for obtaining energy by mineral dissolution. Parts of their EPS may be left behind as footprints. If the location is suitable, they secrete more EPS to enhance attachment. Both possibilities cause the production of artifacts. With 10 mg/L SX reagent the attached cells accumulated, but their profile was not sharp (Figure 11B1). With 50 mg/L SX reagent, accumulation of cells was also observed (Figure 11C1) but less dense than that with 10 mg/L SX reagent. Their profile was sharp, and a thin layer of EPS around the biofilm cells could be clearly imaged. With 50 mg/L SX reagent, no artifacts were produced, indicating that the EPS were solid and non-sticky. Such type of EPS has been observed previously, when biofilms of *Sb. thermosulfidooxidans* had developed on elemental sulfur (Zhang et al., 2015; Li et al., 2020).

Sulfobacilli have a versatile metabolism and can grow autotrophically with Fe^2+^, RISCs, and metal sulfides but also heterotrophically and mixotrophically. They grow best if organic carbon is supplemented, and their autotrophic and heterotrophic growth is often restricted to several transfers (Norris et al., 1996; Karavaiko et al., 2006). Their enhanced growth and biofilm formation with 10 mg/L SX reagent might have benefitted from the degradation of the SX reagent. It is reported that the degradation of LIX984N results in the formation of aldehydes and ketones and other compounds (Shakibania et al., 2020). Thus, it is possible that degradation of the solvent might provide an additional carbon source and support the bacterial sustainable growth. It is unknown why 50 mg/L SX reagent stopped the cells of *Sb. thermosulfidooxidans* from oxidizing Fe^2+^ and caused them to oxidize RISCs. In our previous study we detected that under autotrophic conditions, cells of *Sb. thermosulfidooxidans* mainly grew on RISCs (Li et al., 2020). Perhaps using RISCs is favorable for survival of *Sb. thermosulfidooxidans* under stress. Zhou et al. (2015) studied the expression of critical sulfur and iron oxidation genes and community dynamics during bioleaching of chalcopyrite by moderate thermophiles. They also found that *Sb. thermosulfidooxidans* was the dominant strain during the whole bioleaching process. The gene expression analysis indicated that *Sb. thermosulfidooxidans* was mainly responsible for sulfur oxidation (Liu et al., 2016).

## Conclusion

The inhibitory effects of traces of the SX reagent in the leaching solution on microbial activity during bioleaching of chalcopyrite waste rock are quite different from those noted for incubation with only Fe^2+^- or S°-containing media. In an Fe^2+^-containing medium the SX reagent did not affect growth and Fe^2+^ oxidation of *At. ferrooxidans*. In a S°-containing medium the SX reagent caused the growth of *At. ferrooxidans* in two stages, and the bacteria grow almost as good as without the SX reagent. In the bioleaching process the SX reagent caused delays in proliferation of actively Fe(II)-oxidizing cells of *At. ferrooxidans* and interfered with the bacterial biofilm formation and the oxidization of RISCs, which in turn inhibited bioleaching. In the case of *L. ferrooxidans* the SX reagent prolonged its lag times and caused delays in Fe^2+^ oxidation when it grew in Fe^2+^-containing medium. However, when bioleaching the bacteria only tolerated 10 mg/L SX reagent. SX reagent (50 mg/L) killed the bacteria. In Fe^2+^- or S°-containing medium, the growth of *Sb. thermosulfidooxidans* could not be detected. However, the bacterial growth occurred with chalcopyrite waste rock in the presence of the SX reagent. The bacteria, after adaptation to 10 mg/L SX reagent, performed bioleaching as good as the natural ones, since their growth and biofilm formation were enhanced. With 50 mg/L SX reagent, although *Sb. thermosulfidooxidans* developed a good biofilm, its Fe^2+^ oxidation became inhibited, and the cells started to oxidize RISCs. As a result, bioleaching did not occur. Our study suggests that a removal of the SX reagent after solvent extraction is necessary, and cells of *L. ferrooxidans* can be used as a good indicator of high concentration of SX reagent. Fe^2+^-oxidizing biofilm formation at the initial bioleaching stage is crucial, because there exists a positive relation between biofilm formation and bioleaching. For a newly constructed heap mixing, the fresh ores with some leached ores or adaptation of the microorganisms to the SX reagent and supplementary addition of ferrous iron in the recycled leachate may be helpful to initiate the bioleaching fast and achieve an improved copper extraction.

## Data Availability Statement

The original contributions presented in the study are included in the article/supplementary material, further inquiries can be directed to the corresponding author.

## Author Contributions

XrL, QL, and WS designed the experiments. XhL, YL, JC, ZZ, XrL, and RZ performed the experiments and analyzed the data. QL and WS prepared the manuscript. All authors read and approved the final manuscript.

## Conflict of Interest

The authors declare that the research was conducted in the absence of any commercial or financial relationships that could be construed as a potential conflict of interest.

## Publisher’s Note

All claims expressed in this article are solely those of the authors and do not necessarily represent those of their affiliated organizations, or those of the publisher, the editors and the reviewers. Any product that may be evaluated in this article, or claim that may be made by its manufacturer, is not guaranteed or endorsed by the publisher.
